# Wheat beta-expansin (EXPB11) genes: Identification of the expressed gene on chromosome 3BS carrying a pollen allergen domain

**DOI:** 10.1186/1471-2229-10-99

**Published:** 2010-05-27

**Authors:** James Breen, Dora Li, David S Dunn, Ferenc Békés, Xiuying Kong, Juncheng Zhang, Jizeng Jia, Thomas Wicker, Rohit Mago, Wujun Ma, Matthew Bellgard, Rudi Appels

**Affiliations:** 1 Centre for Comparative Genomics (CCG), Murdoch University, South Street, Perth 6150, Australia; 2 Molecular Plant Breeding Co-operative Research Centre (MPBCRC), Murdoch University, South Street, Perth 6150, Australia; 3 State Agricultural Biotechnology Centre (SABC), Murdoch University, Murdoch University, South Street, Perth 6150, Australia; 4 CSIRO Plant Industries, PO Box 1600, Canberra, Australian Capital Territory 2601, Australia; 5 Key Laboratory of Crop Germplasm Resources and Utilization, MOA/Institute of Crop Sciences, CAAS/The Key Facility for Crop Gene Resources and Genetic Improvement, Beijing 100081, PR China; 6 Institute of Plant Biology, University Zurich, Zollikerstrasse 107, Zurich, CH-8008 Switzerland; 7 Department of Agriculture and Food, Western Australia (DAFWA), 3 Baron Hay Court, Perth, 6151 Australia; 8 Centre for Clinical Immunology and Biomedical Statistics, Murdoch University, South Street, Perth WA 6150, Australia

## Abstract

**Background:**

Expansins form a large multi-gene family found in wheat and other cereal genomes that are involved in the expansion of cell walls as a tissue grows. The expansin family can be divided up into two main groups, namely, alpha-expansin (EXPA) and beta-expansin proteins (EXPB), with the EXPB group being of particular interest as group 1-pollen allergens.

**Results:**

In this study, three beta-expansin genes were identified and characterized from a newly sequenced region of the *Triticum aestivum *cv. Chinese Spring chromosome 3B physical map at the *Sr2 *locus (FPC contig *ctg11*). The analysis of a 357 kb sub-sequence of FPC contig *ctg11 *identified one beta-expansin genes to be *TaEXPB11*, originally identified as a cDNA from the wheat cv Wyuna. Through the analysis of intron sequences of the three wheat cv. Chinese Spring genes, we propose that two of these beta-expansin genes are duplications of the *TaEXPB11 *gene. Comparative sequence analysis with two other wheat cultivars (cv. Westonia and cv. Hope) and a *Triticum aestivum *var. *spelta *line validated the identification of the Chinese Spring variant of *TaEXPB11*. The expression in maternal and grain tissues was confirmed by examining EST databases and carrying out RT-PCR experiments. Detailed examination of the position of TaEXPB11 relative to the locus encoding *Sr2 *disease resistance ruled out the possibility of this gene directly contributing to the resistance phenotype.

**Conclusions:**

Through 3-D structural protein comparisons with *Zea mays EXPB1*, we proposed that variations within the coding sequence of *TaEXPB11 *in wheats may produce a functional change within features such as domain 1 related to possible involvement in cell wall structure and domain 2 defining the pollen allergen domain and binding to IgE protein. The variation established in this gene suggests it is a clearly identifiable member of a gene family and reflects the dynamic features of the wheat genome as it adapted to a range of different environments and uses.

Accession Numbers: *ctg11 *=FN564426

Survey sequences of *TaEXPB11ws *and *TsEXPB11 *are provided request.

## Background

Cereal plant crops are vital to the overall health of the world's population and genome sequencing is an important step in the genetic improvement of crops. While hexaploid wheat (*Triticum aestivum *L.) accounts for nearly one-fifth of the entire world's daily calories [1], the sequencing of its genome has been restricted by high sequencing costs associated with its large genome size (~16,000 Mb) and high (~80%) repetitive content [2]. The published physical map of the largest wheat chromosome 3B [3], which itself is twice the size of the entire rice genome, has allowed researchers to target specific regions that have been identified to contain agronomically important traits such as fungal resistance or grain quality. Projects co-coordinated within the International Wheat Genome Sequencing Consortium (IWGSC) on chromosome 3B aim to tackle the challenges associated with genome sequencing through collaboration, and facilitate the study of significant multi-gene families.

One such multi-gene family found extensively in the wheat and other cereal genomes are the expansins. It has been estimated that the hexaploid wheat genome contains more than 95 expressed members [4], much higher than the rice genome. Expansins belong to a large group of proteins found within the structure of plant cell walls and are considered to be involved in the expansion of cell walls as a tissue grows [5]. The proposed model of expansin action is that these proteins modify the cell-wall matrix to enable growth and development of plant cells [6-8] and, as a result, expansins have been implicated in providing resistance to certain diseases [9]. The latter was of particular interest because it was located in a region of the wheat genome being sequenced in order to define disease resistance genes in the region. Expansins were originally isolated from cucumber seedlings and have 'acid growth' characteristics, where they can stimulate cell enlargement in the response to acid pH [10]. Expansins have now been reported in many plants such as cotton [*Gossypium hirsutum*; [11,12]], tomato [*Lycopersicon esculentum*; [13]], Arabidopsis [*Arabidopsis thaliana*; [14,15]] and pea [*Pisum sativum*; [16]]. cDNA clones have also been isolated from wheat [4,17-19] and barley [*Hordeum vulgare*; [20]].

The multi-gene expansin family can be divided up into two main groups, namely, α-expansin (EXPA) and beta-expansin proteins (EXPB), which share very limited (~20%) amino acid similarity even though both are associated with cell-loosening activity [6]. The beta-expansin proteins were originally viewed exclusively as group 1 pollen allergens but are now considered to be important in cell wall changes during growth in vegetative tissues of grasses and dicotyledon plants [18], most notably in development and growth zones of tissues such as roots [20,21]. The group 1 pollen allergen domain-containing beta-expansin proteins are highly expressed in mature pollen of grass species and are thought to have a role in pollen tube penetration [14,22]. Pollen-triggered allergic reactions (e.g. hayfever and seasonal asthma) affect up to 25% of adults in industrialized nations [23]. Group 1 allergens bind to group 1 specific IgE antibodies [24] and a well studied example is the pollen allergen, Phl p 2 in timothy grass (*Phleum pratense*) where a specific protein domain has been identified as a binding site [25].

A previous study analysed the sera of patients that had undergone positive double blind, placebo-controlled food challenge to hexaploid wheat and identified the gene *TaEXPB11 *as one of 12 genes that encoded proteins binding to the IgE from wheat sensitive patients [19]. In the present study, we present the identification of three beta-expansin genes through a genome sequencing study of the short-arm of chromosome 3B in hexaploid wheat (*T. aestivum *cv Chinese Spring). Structural analyses of the three *TaEXPB11*-Chinese Spring variants indicate a localised gene duplication producing non-coding gene copies. Re-analysis of recombinant lines using specific markers from the expansin genes indicated that they were not linked to the Sr2 resistance phenotype of wheat cv. Hope, arguing against an involvement in the resistance. Comparative sequence analysis was also undertaken on selected cultivars and a *T. spelta *wheat accession demonstrating changes within the gene-coding sequence of *TaEXPB11 *produced protein structural changes. Expression of *TaEXPB11*, experimentally and within EST databases, was also assessed. Our study provides novel insights into the structure of beta-expansins and their variation across different wheat genomes.

## Results

### *Ctg11 *wheat genome sequencing

**Figure 1:**
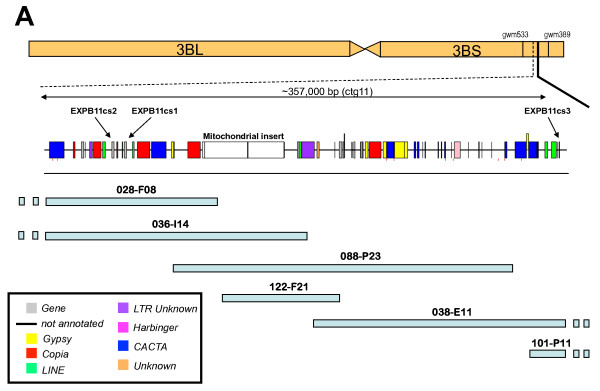
**Genomic location, bacterial artificial chromosome (BAC) clone map and sequence annotation of 357 kb from the hexaploid wheat (*T. aestivum *cv Chinese Spring) *ctg11 *genomic sequence of chromosome 3B containing three beta-expansin genes**.

The physical map of chromosome 3BS of hexaploid wheat (cv. Chinese Spring and cv. Hope) has been compiled through DNA fingerprinting of the flow-sorted chromosome 3B BAC library and anchoring BAC contigs to genetic maps [4,26]. The shotgun sequencing of BACs located within a minimum tilling path of ca 1.3 Mb across the *Sr2 *locus on chromosome 3BS [26] was carried out to identify possible candidate genes for this disease resistance locus (R. Mago et al. 2010, *in preparation*). The BAC clones characterized in this study, and related work (Choulet et al. 2010; Wicker et al. *in preparation*), indicated the presence of an active expansin gene which was a candidate for the *Sr2 *locus because, as a cell wall component, the product of the gene has been associated with disease resistance [27]. A 357 kb region the *ctg11 *genome sequence containing three beta-expansin genes was annotated in detail for the present study (Figure 1)

The 357 kb *ctg11 *sequence contained nine complete or partial gene-coding regions (Table 1) giving it a gene density of one gene per 44.6 kb. The sequence contained 133,134 bp (33.2%) repetitive DNA in the form of transposable elements (TEs) and a 51,666 bp mitochondrial genome insert. The presence of the mitochondrial DNA insert in normal genomic DNA has been confirmed by assaying the genomic sequence for mtDNA-chromosomal DNA junctions (data not shown). Further genome sequencing was carried out using 454-technology sequencing of BAC clone 3B_036_I14 (Zhang, J. and Kong, XY., unpublished), which contained two full-length beta-expansin genes and the mitochondrial insert, in order to confirm the presence of the mitochondrial insert and improve the sequence assembly in some regions. Additional BAC sequencing was also carried out on the syntenic *ctg11 *region of wheat cv. Hope (R. Mago et al. 2010, *in preparation*).

**Table 1:** Gene-coding annotation of the 357,000 bp sub-sequence of *ctg11*

					Rice Genome	Wheat EST Analysis		
**Gene**	**Length (nt)**	**# Exons**	**Predicted protein length (aa)**	**Rice Chr**.	**Top Rice protein hit (MSU Rice annotation version 5)**	**Wheat EST**	**E-value**	**Wheat Unigene set**

EXPB11cs2	1233	3	275	3	LOC_Os03g01270	CJ674809	1e-178	Ta.31031
EXPB11cs1	1342	3	290	3	LOC_Os03g01270	CJ674809	2e-105	Ta.31031
FMO1	1720	4	469	7	LOC_Os07g02140	CK207166	9e-124	Ta.32721
RGA1	3037	3	553	1	LOC_Os01g36640	CJ948865	2e-78	Ta.41149
WDL1	1841	7	394	11	LOC_Os11g38010	CJ661904	3e-104	Ta.57217
DPC1	2707	3	230	1	LOC_Os01g25880	CK212399	0.0	Ta.55136
UTG1	1470	1	490	11	LOC_Os11g38650	BU100894	6e-152	Ta.51207
ADP1	3805	6	308	5	LOC_Os05g04860	CK210567	2e-66	Ta.4597
EXPB11cs3	291	1	97	3	LOC_Os03g01270	BQ608206	6e-129	Ta.31031

### Structural characterisation and validation of three beta-expansin genes in *ctg11*

**Figure 2:**
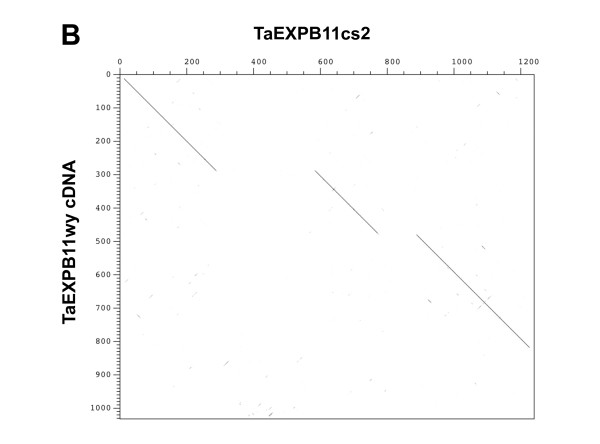
**Dot matrix to show the nucleotide sequence comparison between the *TaEXPB11cs2 *and *TaEXPB11wy *cDNA sequence (isolated from wheat cv. Wyuna **[19]).

The 357 kb subsequence of *ctg11 *was masked for repetitive elements by running RepeatMasker (Smit and Green, http://www.repeatmasker.org/) using the Triticeae repeat sequence (TREP) database http://wheat.pw.usda.gov/ITMI/Repeats/. Gene predictions were then searched against a protein subsection of the TREP database using BLASTP to ensure no repetitive predicted proteins were included in the analysis. Two highly similar gene models were predicted and found within 7,418 bp of each other with a third similar truncated copy located over 320 kb away (Figure 1). Annotation of the genomic sequence identified that the three copies of sequences were related to the beta-expansin family [27], with the second copy of the three genes being very similar to a published *TaEXPB11 *cDNA (Figure 2) [19]. The published *TaEXPB11 *sequence was recovered from an endosperm cDNA library from wheat cv. Wyuna [28].

The coding regions of the beta-expansin that has the highest sequence similarity to the published *TaEXPB11 *cDNA has 6 amino acid differences (98.2% nucleotide sequence similarity) resulting from single nucleotide polymorphism (SNP) differences; these SNPs are not unexpected considering the published *TaEXPB11 *derived from wheat cv. Wyuna and the genomic sequence from wheat cv. Chinese Spring. We propose to name this gene *TaEXPB11cs2 *with the names of copies located upstream being *TaEXPB11cs1 *and *TaEXPB11cs3 *respectively. The *TaEXPB11 *from wheat cv. Wyuna [19] will be referred to as *TaEXPB11wy *in this study.

*TaEXPB11cs1 *had a lower sequence similarity (73.36%) to the *TaEXPB11wy *cDNA and a slightly lower similarity to the identified rice homolog *OsEXPB7 *(63% compared to 64.83% in *TaEXPB11cs2*). The *TaEXPB11cs3 *gene was very closely related to *TaEXPB11wy *except for the fact that the gene is truncated, containing only one exon (97 amino acids or 35.4% of the full-length *TaEXPB11 *gene). The coding regions of all the genes were found to have high sequence similarity (>80% at the nucleotide level) to a full-length wheat cDNA from a recently published database of 11,902 full-length wheat cDNA sequences (KOMUGI; http://www.shigen.nig.ac.jp/dnadb/index.jsp).

**Figure 3:**
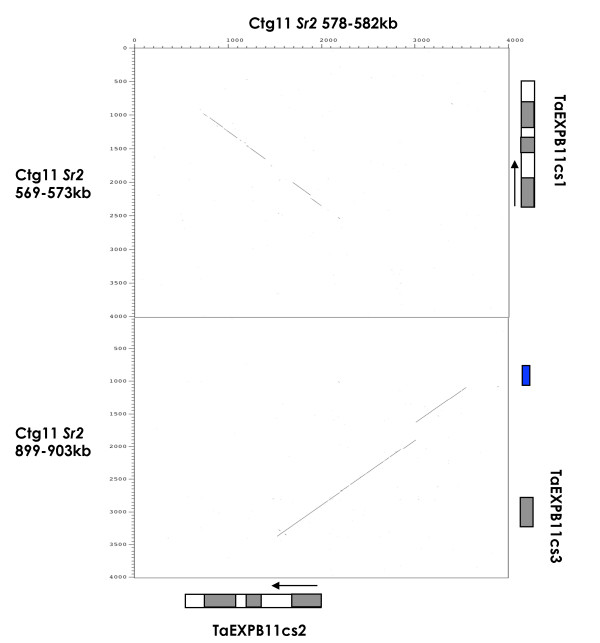
**Dot-matrix plot of 4 kb of the genomic sequence surrounding *TaEXPB11cs2 *(horizontal axis) against 4 kb of the two other beta-expansin genes (*TaEXPB11cs1 *and *TaEXPB11*c3) found within the wheat cv**. Chinese Spring assembled *ctg11 *genome sequence. The exon regions of each gene have been annotated. The blue box indicates a small 269 bp CACTA DNA transposon located near the *TaEXPB11cs3 *gene fragment.

Comparing the nucleotide sequence to *TaEXPB11wy *identified the intron-exon structure of *TaEXPB11cs2 *(Figure 2). *TaEXPB11cs1 *and *TaEXPB11cs2 *(compared in Figure 3) both have three exons with intron 1 (348 bp in *TaEXPB11cs2*) being more than twice the size of intron 2 (122 bp), while the truncated gene *TaEXPB11cs3 *does not contain any introns. *TaEXPB11cs1 *and has a 42 bp insertion at position 90-132, within the first exon of the gene when compared to *TaEXPB11cs2*. This insertion in *TaEXPB11cs1 *contains a stop codon which suggests that it is probably a pseudo-gene.

The intron 1 sequences from *TaEXPB11cs1 *and *TaEXPB11cs2 *were compared to identify whether or not they represented recent duplications. Both genes sequences have a 92% sequence similarity over 1,514 bp. Outside the coding sequence of the genes, 28 bp of sequence 5' from the start codon and 26 bp 3' from the end codon, are conserved between *TaEXPB11cs1 *and *TaEXPB11cs2*. The comparison in Figure 3 between *TaEXPB11cs2 *and *TaEXPB11cs3 *demonstrates that the first intron is found to be well conserved between both sequences, as well as 880 bp of genomic sequence 5' to the start of the first exon. The beta-expansin genes amplified from the genomic DNA of a number of varieties of wheat (see later), showed that the respective *TaEXPB11cs2*-type genes were readily distinguished from *TaEXPB11cs1 *based on sequence divergence in intron 1. Intron 2 was not as diagnostic for distinguishing the *TaEXPB11cs1 *and *TaEXPB11cs2 *gene categories. Exon 3 in *TaEXPB11cs1 *showed a characteristic 35 bp insertion that was not present in any of the other TaEXP11cs type genes.

The ages of both *TaEXPB11cs1 *and *TaEXPB11cs3 *(proposed duplicates of *TaEXPB11cs2*) were estimated using methods used to date LTR retrotransposons [29]. The nucleotide sequence used for comparison between *TaEXPB11cs2 *and the two proposed gene duplications was from the start codon of each gene or gene fragment (in the case of *TaEXPB11cs3*), covering all of exon 1 and part of the intron 1 sequence (452 bp in total). The *TaEXPB11cs1 *duplication was identified to have duplicated 5.69 million years ago (MYA) with a standard deviation of 0.77 MYA, while the *TaEXPB11cs3 *duplication was much younger at only 1.59 MYA (standard deviation of 0.38 MYA.

**Figure 4:**
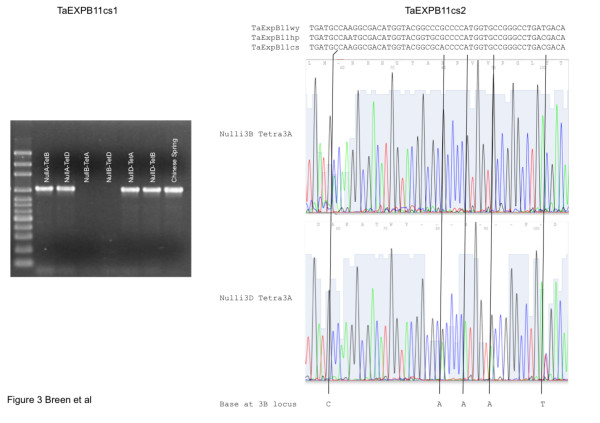
**Nulli-Tetra lines for the group 3 chromosomes in Chinese Spring genetic stock lines analysed for occurrence of *TaEXPB11cs1 *(left panel) and *TaEXPB11cs2 *(right panel)**. A (left panel). For the chromosome assignment of *TaEXPB11cs1*, PCR products assaying exon 1 with one of the primers located in the insertion that characterizes *TaEXPB11cs1 *(see Material and Methods) were analysed on 2% agarose gels and stained with SYBR Green. The molecular markers are a 100 bp ladder (Axygen) and the product size of just over 1000 bp was as expected from the genome sequence (1027 bp). B (right panel). For the chromosome assignment of *TaEXPB11cs2 *(right panel), SNPs in exon 1 that differentiated the genes on chromosome 3A, 3B and 3D were assayed by direct sequencing of PCR products from primers that amplified a common section of this exon. All possible Nulli-Tetra combinations were assayed and the sequences compared to the respective sequence from the *TaEXPB11wy*, *TaEXPB11hp *and *TaEXPB11cs2 *genes (top of figure) in order to assign SNPs to particular chromosomes in the Nulli-Tetra combinations.

Validation of sequence structure for the expansin region assembled in Figure 1, in genomic DNA was carried out for *TaEXPB11cs1 *and *TaEXPB11cs3 *because their structure was unusual and it was important to ensure that changes had not occurred during the BAC cloning process. For *TaEXPB11cs3*, primers were designed to amplify DNA fragments from the borders of the regions that had a high similarity to *TaEXPB11cs2 *and these were predicted to generate fragments 686 bp and 856 bp long. The respective fragments generated had approximate sizes (estimated by agarose gel electrophoresis, data not shown) of ca 800 bp, in reasonable agreement with the expected sizes. The unusual structure of *TaEXPB11cs1 *(insert in exon 1 and a small insert in exon3) was shown to exist in genomic DNA by amplifying a DNA fragment using primers from within the respective insertions and which were predicted to generate a 1027 bp fragment. The results (Figure 4) indicated that the fragment obtained was slightly larger than 1000 bp using agarose gel electrophoresis, in agreement with the expected size. The analysis of Nulli-Tetra stocks of wheat showed that *TaEXPB11cs1 *was present only on chromosome 3B since the PCR product was missing when chromosome 3B was missing from the wheat line analysed (Nulli3B-Tetra3A and Nulli3B-Tetra3D lines; Figure 4).

The transcribed gene, *TaEXPB11cs2*, was shown to be present on chromosomes 3A, 3B and 3D using the same Nulli-Tetra stocks illustrated in Figure 4, generated PCR products of the same size from the three chromosomes (data not shown). Several SNP differences between *TaEXPB11cs2 *on chromosomes 3A, 3B and 3D were detected by sequencing a genome PCR product directly (see for example Figure 4). Sequencing of the PCR products from the respective Nulli-Tetra lines allowed the assignment of the base pair differences to a chromosome. In Figure 4, an example is presented for exon 1 where several base positions show a mixed base-call in the lower panel (direct sequencing of genome DNA PCR product) where chromosome 3B is present (example shown is a genetic stock where only chromosome 3D is missing). In the genetic stocks where 3B is missing (Nulli 3B) the mixed base-call is resolved indicating that the base which is removed in the top panel is located on the chromosome 3B site for expansin *EXBP11*. Although only two examples are shown in Figure 4, all possible Nulli-Tetra combinations for 3A, 3B and 3D were analysed. Based on SNP analyses, the 3B gene was more similar to the published *TaEXB11 *than the genes on 3A and 3D but the relationship was not unambiguous because of the background of SNPs expected between wheat cv. Chinese Spring (source of the genome sequence) and cv. Wyuna (source of the published *TaEXPB11 *cDNA).

### Comparative sequence analysis in the beta-expansin gene sequence from selected wheat species

**Figure 5:**
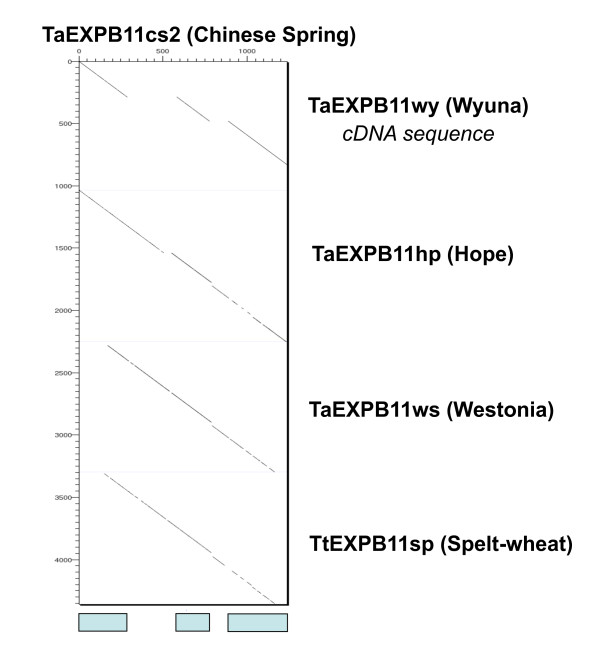
**Pair-wise sequence comparison of the *TaEXPB11 *genomic sequences of wheat cultivar Chinese Spring and an example of the 'survey' sequences (spelt-red) carried out in this study on different wheat cultivars and spelt species**. The three exons of *TaEXPB11cs2 *are indicated below the figure (pale blue boxes, exon 1, 2 and 3 from left to right).

The beta-expansin gene PCR products from wheat cv. Westonia, cv. Hope and cv. Wyuna (published cDNA sequence; AJ890019), and a *T. spelta *line were compared to *TaEXPB11cs2 *in Figure 5. The cv Westonia and *T. spelta *survey sequences were recovered from genomic DNA using primers positioned just inside the first exon and 60 bp before the end of the gene in order to amplify exon and intron sequences. The resulting sequences amplified from the DNA samples was ~1,000 bp in length and were specific as judged from direct sequencing of the PCR product. In the genomic BAC sequencing of wheat cv. Hope (R. Mago et al. 2010 *in preparation*), an ORF from the sequence was identified and named *TaEXPB11hp *as it contained the three predicted exons and was shown to have high sequence similarity to *TaEXPB11wy *as well as *TaEXPB11cs2*. Exon 3 of this *TaEXPB11hp *gene was truncated due to a point mutation causing a premature end to the coding sequence. Overall the nucleotide sequence similarity of *TaEXPB11hp *and *TaEXPB11cs2 *was 90%. The wheat cv. Westonia beta-expansin (*TaEXPB11ws*) and *T. spelta *(*TaEXPB11sp*) accessions showed 96.5% sequence similarity.

### Transcription of the TaEXPB11cs genes

Assessing the data sets available from the NCBI Unigene EST profile (Ta.31031) indicated that two *TaEXPB11 *ESTs were present in libraries comprising 960,174 EST clusters. The ESTs were identified as being expressed from seed (6 transcripts per million or TPM) and flower (15 TPM). No evidence could be found for the expression of *TaEXPB11cs1*, using the insertion sequence in exon 1 that is unique to this gene, as a probe.

**Figure 6:**
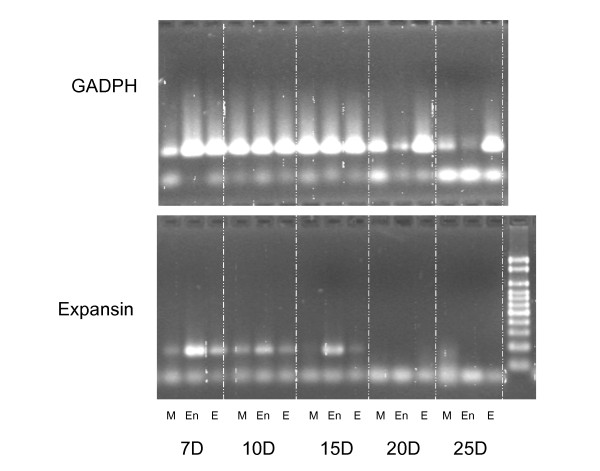
**Gel analysis show samples from the RT-PCR (2% agarose gel) of the internal control GADPH (A, top panel) and the first exon of TaEXPB11cs2 (B, lower panel)**. Material was analysed 7, 10, 15, 20 and 25 days post anthesis and included maternal tissue from the developing grain (M), endosperm (En) and embryonic (Em) tissues of wheat cv Cranbrook.

The endosperm expression of *TaEXPB11cs2 *was confirmed by RT-PCR (Figure 6). The tissue analysed was from developing grain with endosperm and embryo tissues separated by hand. The pericarp tissue was retained as an example of maternal tissue. In a study of the time course of expression, using tissues collected 7, 10, 15, 20 and 25 days post anthesis, strong expression was found for the three tissues at all stages of development. Quantitative analyses of the RT-PCR data indicated that the relatively lower expression for the maternal tissue suggestive in Figure 6 was not significant (data not shown). No evidence for the transcription of *TaEXPB11cs1 *could be found in these RT-PCR experiments consistent with the analysis of available EST databases.

### Protein domain characterisation

**Figure 7:**
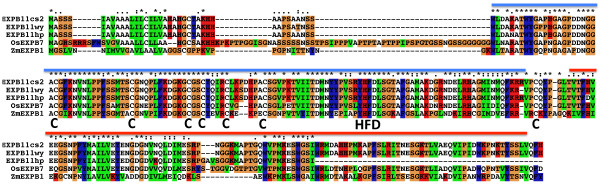
**Multiple sequence alignment of two full-length beta-expansin genes found in wheat genomic sequences of chromosome 3B (*TaEXPB11cs1 *and *TaEXPB11cs2*) compared to the *TaEXPB11 *cDNA, rice homolog OsEXPB7 and maize Zea M 1 using the ClustalX program **[44]. The blue (domain 1) and red (domain 2) lines above the sequence indicate the different domains and the signature EXPB motifs (the 'HFD' and conserved cysteine residues) are indicated below the sequence.

The well-characterised maize beta-expansin gene (*EXPB1 *in *Zea mays*) purified from maize pollen, and its crystal structure [30] was used to validate the two protein domains commonly identified in expansins, within the full-length *TaEXPB11*cs*2 *and *TaEXPB11hp *genes. Both genes contained conserved cysteine residues that create disulphide bonds between the domain folds as well as the characteristic 'HFD' motif that is the catalytic site of the distantly related family-45 endoglucanases (domain 1, GH45) [27] (Figure 7). Domain 1 is a lipoprotein A (RlpA)-like double-psi beta-barrel family domain (PF03330) commonly found at the N-terminus of pollen allergens. Domain 2 (Figure 7) is a Pollen_allerg_1 family (PF01357), grass type-2 pollen allergen domain originally characterised in timothy grass (*P. pratense*) [31]. The conserved residues in domain 2 outlined in [32] could also be identified although, when compared to the maize *EXPB1 *sequence, its amino acid sequence is shown to be more diverged (44% identity over 99 amino acids) compared to domain 1 (66% over 125 amino acids). As mentioned previously (and shown in Figure 7), domain 2 is truncated in *TaEXPB11hp *due to a stop codon terminating the coding sequence.

## Discussion

Expansins, a cell-wall loosening class of proteins, are a multigene family found in grass genomes that are considered to play important roles in growth and development in wheat [17]; EST mining has estimated that there exist at least 30 and 65 alpha- and beta-expansins, respectively [4] in wheat. The higher beta-expansin gene estimate compared to alpha-expansins within wheat is consistent with the estimates in other grasses such as maize [33]. Different expression characteristics suggest that the two expansin classes play different functional roles within the cell wall structure [5].

In the present study we identified and characterised three beta-expansin genes from wheat cv. Chinese Spring located in a ~357 kb region of chromosome 3BS. Only one of these genes was found to be the *TaEXPB11 *gene, coding for an IgE binding protein identified from the sera of patients that had undergone positive double-blind, placebo-controlled food challenge to wheat [19]. Sequence analysis of specific PCR products using *TaEXPB11 *primers from the wheat cultivars and a *T. spelta *wheat accession validated the identification of the wheat cv. Chinese Spring variant of *TaEXPB11 *cDNA [18], originally obtained from wheat cv. Wyuna [28]. It is proposed that sequence variants of *TaEXPB11 *be designated *TaEXPB11wy*, *TaEXPB11*cs, *TaEXPB11ws*, *TaEXPB11hp*. The high sequence similarity between the *TaEXPB11 *cDNA and *TaEXPB11cs2 *provided good evidence for assigning the two sequences to be alleles of the same gene. Even though a large family of expansin genes has been reported in wheat [4,17], the primers used to assay the transcripts (Figure 5) and gene locations did not provide any evidence of the presence of other copies of this particular gene elsewhere in the genome. The primers are evidently specific enough to assay only the *TaEXPB11 *gene category on homoeologous locations on chromosome 3A, 3B and 3D.

Utilizing SNPs identified in the present study, expansin was ruled out as a candidate gene for *Sr2 *resistance because re-examination of recombinant Chinese Spring-Hope lines studied by Kota et al [34] identified a recombination event between the *Sr2 *phenotype and *TaEXPB11hp *(R. Mago unpublished). We note that in the region examined, wheat cv Hope (the source of *Sr2*) is missing the mitochondrial DNA insert found in wheat cv Chinese Spring. This mitochondrial DNA segment encodes a gene *nad7*, which is a subunit of NADH dehydrogenase that is active in producing reactive oxygen species. Although this is not genetically linked to the *Sr2 *resistance gene, its close proximity may indicate it is part of a gene network controlling levels of active oxygen [35] (a generally accepted feature of resistance genes).

The *TaEXPB11cs *genes identified from the genome sequencing of the *ctg11 *contig on the small arm of wheat chromosome 3B could be characterized in detail. The *TaEXPB11cs3 *gene fragment contained only one coding exon and showed an extremely high sequence similarity to *TaEXPB11cs2*. This sequence similarity extended not only over its coding sequence but also the 880 bp 5' upstream from the start of the coding sequence of its only exon, as well as part of the sequence corresponding to the first intron sequence. The high sequence similarity over the coding and non-coding regions of the two sequences indicated that *TaEXPB11cs3 *is a very recent duplication of *TaEXPB11cs2 *with an estimated duplication age of 1.59 MYA. Direct genome analysis confirmed the existence of *TaEXPB11cs3*. The neighbouring CACTA DNA transposon '*Caspar*' found within a base pair of the duplicated *TaEXPB11cs3 *fragment suggests the possibility that this genomic duplication was mediated by this TE or was the result of a TE-mediated recombination event [29]. While CACTA DNA transposons have not yet been shown to be involved in gene fragment duplication and the creation of chimeric genes, repetitive elements such as pack-MULEs found in multiple copies within the rice genome, have been shown to capture gene fragments and other genomic DNA to create chimeric ORFs [36].

The *TaEXPB11cs1 *gene appeared to be an older duplication of *TaEXPB11cs2 *with an estimated age of the duplication being 5.69 MYA. There was significant conserved nucleotide sequence either side of the gene coding sequences and a high level of sequence similarity between *TaEXPB11*cs1 and *TaEXPB11cs2 *intron2 sequences. The characteristic insertions within *TaEXPB11cs1 *(Figure 3) were validated by direct genome PCR and the pseudo-gene was shown to exist only on chromosome 3B using Nulli-Tetra mapping lines of wheat.

At the level of the SNP analysis it is clear that very similar genes exist as homoeologues on chromosomes 3A, 3B and 3D. Each of these homoeologous genes could be contributing to the mRNA assayed in Figure 6. The SNP analysis in Figure 4 is consistent with *TaEXPB11cs2 *representing the cDNA identified as *TaEXPB11*.

A well-characterised maize beta-expansin gene (*EXPB1 *in *Zea mays*) was used as a comparison with *TaEXPB11cs2*, to identify the particular motifs that are conserved between the protein domains. The three wheat beta-expansin genes, shown in Figure 7, contained the conserved cysteine residues that form disulphide bonds between the domain folds and the 'HFD' motif that is common in the catalytic site of the distantly related family-45 endoglucanases (GH45) [30]. They also contained many conserved residues in domain 2 outlined in [31,32], but when compared to the maize EXPB1 sequence, this allergen domain amino acids sequence is shown to be more diverged (44% identity over 99 amino acids) compared to domain 1 (66% identity over 125 amino acids), (Figure 7). Similar folding patterns were identified in *TaEXPB11hp *except that the amino acid sequence in the pollen allergen domain was largely missing.

## Conclusions

Sequence analysis and annotation of 357 kb of chromosome 3B genomic sequence identified three beta-expansin genes, one of which was identified to be *TaEXPB11*, originally from a cDNA identified from wheat cv. Wyuna. Through the analysis of intron sequences of the three wheat cv Chinese Spring genes, we propose that two of these beta-expansin genes are duplications of the *TaEXPB11 *gene. Comparative sequence analysis with two other wheat cultivars (Westonia and Hope) and a *T. spelta *accession validated the identification of the wheat cv. Chinese Spring variant of *TaEXPB11*. EST and RT-PCR experiments confirmed the expression in maternal and grain tissues. The variation established in this gene suggests it is a clearly identifiable member of a gene family and reflects the dynamic features of the wheat genome as it adapted to a range of different environments and uses.

## Methods

### Wheat BAC sequencing

The sequencing of 20 *T. aestivum *cv. Chinese Spring BACs from *ctg11 *(*Sr2 *locus, http://urgi.versailles.inra.fr/cgi-bin/gbrowse/wheat_FPC_pub/) was carried out using a BAC-by-BAC shotgun method at 6× to 10× sequencing coverage (Wicker et al. 2010 *in preparation*) as well as additional 454 sequencing. The genome sequencing of wheat cv. Hope over the syntenic *Sr2 *region was carried out using the same BAC-by-BAC approach with sequencing carried out at 10× coverage from chromosome 3B and cultivar Hope-specific BAC library (R. Mago et al. 2010 *in preparation*).

*E. coli*-DNA-free BAC DNA were extracted with Qiagen Large-Construct Kit (QIAGEN, Cat. No. 20021) and mechanically sheared with HydroShear as recommended by ABI applied biosystems https://products.appliedbiosystems.com/ab/en/US/adirect/ab?cmd=catNavigate2&catID=604432, generating a concentrated smear ~3-5 kb in length. The sheared fragments were blunt ended with mung bean nuclease and dephosphorylated with Shrimp Alkaline Phosphatase (SAP). The short fragments were then tailed with A by PCR using standard procedures. Fragments ranging from 3-5 kb in size were isolated and ligated into a pCR4-TOPO vector and transformed into TOP10 electrocompetent cells (Invitrogen, Cat. No. K4580-01). The clones were sequenced from both directions with T3 and T7 primers using BigDye3.1 termination chemistry and run on an ABI Prism 3730 XL capillary sequencer (Applied Biosystems, Foster City, Calif., USA). Base calling, quality assessment and sequence assembly were carried out using the PHRED/PHRAP [37]. Gaps were filled by designing PCR primers located on the nearest random clone to the sequence gap. Sequencing was then performed using primer walking with additional dGTP mix and DMSO in the sequencing reaction system.

### Sequence Analysis and Annotation

Repetitive DNA analysis was carried out using RepeatMasker (Smit et al. 1996-2004, http://www.repeatmasker.org) and local alignment searches using BLAST [38] against the Triticeae repetitive element (TREP) database http://wheat.pw.usda.gov/ITMI/Repeats/, gene models were identified by the use of FGENESH http://www.softberry.com/, GENSCAN [39] and GlimmerHMM [40]. Sequence homology searches were carried out using BLAST and protein domains were identified by searching the Pfam protein family database [41] as well as the conserved domains database at NCBI [42]. InterProScan [43] was run against the InterPro protein domain database [44], which also includes a signal peptide and Trans-membrane search. Sequence comparisons were carried out using DOTTER [45] and multiple sequence comparisons were carried out using the CLUSTALX [46]. Graphical display of the sequence map was produced using WICKERsoft (T Wicker, pers. comm.).

Dating of Chinese Spring gene duplications [29] were carried out by comparing two sequences using WATER (EMBOSS; http://www.ebi.ac.uk/Tools/emboss/), using a gap creation penalty of 30 and a gap extension penalty of 0.1 parameters. WICKERsoft scripts (T. Wicker, pers. comm.) were used to identify transversions (Tv) and transitions (Ti) between the two sequences, which were then used with a base-pair substitution rate of 1.3 × 10^-8 ^[47] to identify the age of the two sequences.

### Plant material and analysis

DNA from the standard Nulti-Tetra 'Chinese Spring' wheat stocks [48] (kindly provided by J Raupp, Wheat Genetics Resource Centre, Kansas State University) were used for assigning homoeologous locations (3A, 3B or 3D) for expansin genes. The DNA from these genetic stocks was amplified by primers designed from the genome sequence. Deletion lines and all other wheat varieties were kindly provided by Ms F Drake-Brockman (Department of Agriculture and Food, Western Australia). The *Triticum spelta *line 2255 was originally obtained from the Australian Winter Cereals Collection (Tamworth).

For RT PCR, wheat lines were grown in a glasshouse and heads tagged at anthesis. Developing grain was collected at 7, 10 and 15 days post-anthesis and three tissue types collected. After removing the embryo (one tissue), the endosperm was squeezed out (second tissue) and the remaining maternal tissue formed the third tissue that was collected. Tissue samples were frozen on dry ice and total RNA was extracted from approximately 100 mg of frozen seed tissue using the TRIzol reagent (Invitrogen). The extracted RNA was quantified using a Nanodrop ND-1000 Spectrophotometer before being used for cDNA synthesis. The cDNA was synthesised from 1 ug of total RNA using the High Capacity cDNA Reverse Transcription Kit (Applied Biosystems). Changes in the expression of expansin in seed tissue were examined using real time RT-PCR. The housekeeping gene GAPDH was used as an internal standard in the RT-PCR. Each sample tested was completed in triplicate and mRNA expression levels were quantified using a Corbett RG3000 (following manufacturer instructions, using the delta-CT procedure). PCR amplification was performed in a 20 μl reaction volume containing 10 μl of Power 2 × Power SYBR Green PCR Master Mix, 1 μl of each primer (10 μM) and 1 μl of cDNA. Cycling conditions 95°C for 10 min followed by 40 cycles of 95°C for 10 sec and 57°C for 1 min. The primer sequences are listed in Table 2.

**Table 2:** RT-PCR primers used for expression analysis of *TaEXPB11cs2*-domain 2 and a GAPDH control

Exp Dom2 - For	GAGTCGTGGGGTTCCATCT
Exp Dom2 - Rev	AACTGGACGAGGGAGCTGT
Exp Front - For	GATCCTCTGCATCCTCGTC
Exp Front - Rev	GGGTGGTAAGTTGACGTTCT
Exp InDel - For	CACCAAAAAGCCTCCCTAC
Exp InDel - Rev	AGGTCATGGCAGAGAAGG
GAPDH - For	CGAAGCCAGCAACCTATGAT
GAPDH - Rev	CAAAGTGGTCGTTCAGAGCA

### Protein modelling

SignalP [49] was used to identify signal peptide sequences. Protein models of the expansin genes were created by comparing the amino acid sequence to a closely related expansin sequence using MODELLER [50] and viewing the protein databank file (PDB) output using iMol http://www.pirx.com/iMol/.

## Authors' contributions

All authors read and approved the final manuscript.

JB, TW, MB, DD, RA: assembly of the genome sequence and annotation of genes and TEs

RM: identification of BACs from chromosome 3B

JJ, XK, JZ: sequencing of BAC clones

DL, WM: mapping of ISBPs to wheat genetic map and RT PCRs

FB: interpretation of data related to the different wheat lines studied
